# A285 COMPARISON OF ADVERSE OUTCOMES IN PATIENTS WITH COMBINED INFLAMMATORY BOWEL DISEASE AND PRIMARY SCLEROSING CHOLANGITIS VERSUS ISOLATED PRIMARY SCLEROSING CHOLANGITIS

**DOI:** 10.1093/jcag/gwad061.285

**Published:** 2024-02-14

**Authors:** M Dahiya, H Bedi, A Fetz, E Yoshida, H Ko, B Salh, D Chahal

**Affiliations:** University of British Columbia, Vancouver, BC, Canada; University of British Columbia, Vancouver, BC, Canada; University of British Columbia, Vancouver, BC, Canada; University of British Columbia, Vancouver, BC, Canada; University of British Columbia, Vancouver, BC, Canada; University of British Columbia, Vancouver, BC, Canada; University of British Columbia, Vancouver, BC, Canada

## Abstract

**Background:**

Primary sclerosing cholangitis (PSC) results in progressive inflammation and fibrosis of bile ducts causing chronic cholestasis. PSC is associated with an increased risk of malignancy [hepatobiliary and colorectal cancers (CRC)] compared to the general population, in addition to an increased risk of developing end-stage liver disease, often requiring liver transplantation at a relatively young age. The increased risk of adverse outcomes confers an increased risk of premature death of patients with PSC. PSC is commonly associated with IBD, particularly ulcerative colitis (UC), which in itself carries a higher rate of CRC. A study of the impact of PSC with IBD on development of cancer and survival outcomes is imperative.

**Aims:**

To compare the risk of cancer, liver transplantation, and mortality in PSC-IBD versus isolated PSC.

**Methods:**

Retrospective data from PSC patients from two hospital sites in Vancouver, British Columbia was analyzed to compare the relative risk of death, transplantation, and malignancy in PSC-IBD versus isolated PSC.

**Results:**

169 patients with PSC were included in the analysis [mean age of diagnosis of PSC 37 (SD 16.92) years, 41.4% (70) female]. Out of the 169 patients with PSC, 102 had IBD [29.4% (30) with Crohn’s Disease (CD); 70.6% (72) with UC]. The mean age of IBD diagnosis was 28.96 (SD 14.46) years. Death occurred in 31 [64.5% (20) with IBD; RR 1.19, 95% CI 0.61-2.32, p=0.60] patients, 35 [57.1% (20) with IBD; RR 0.88, 95% CI 0.48-1.59, p=0.66] patients developed cancer, and 33 [66.7% (22) with IBD; RR 1.31, 95% CI 0.68-2.52, p=0.41] patients required liver transplantation. The cause of death was malignancy in 15 [53.3% (8) with IBD] patients, liver failure in 5 [80% (4) with IBD] patients, sepsis in 2 [100% (2) with IBD] patients, and unknown cause of death in 9 [77.7% (7) with IBD] patients. For malignancy types, 16 [68.75% (11) with IBD] patients had cholangiocarcinoma (CCA), 4 [50% (2) with IBD] had CRC, 2 [50% (1) with IBD] had gallbladder cancer, 1 [0% (0) with IBD] had pancreatic cancer, 1 [0% (0) with IBD] had esophageal cancer, and 11 [54.5% (6) with IBD] had other malignancies not involving the hepatobiliary or gastrointestinal system. Of the patients who died due to malignancy, 73.3% (11) had CCA.

**Conclusions:**

Although we do not observe any statistically significant difference in risk of cancer, transplantation, or all-cause mortality in patients with PSC-IBD when compared to isolated PSC, we do observe that 59% of all-included patients experienced one or more adverse outcome. It is important for clinicians to recognize the high rates of adverse outcomes in PSC-IBD and isolated PSC patients, potentially warranting earlier or more frequent screening for outcomes such as malignancy and liver dysfunction.

**Funding Agencies:**

None

